# Le repositionnement incisif: quel impact sur la position céphalométrique des points A et B?

**DOI:** 10.11604/pamj.2022.41.209.26071

**Published:** 2022-03-14

**Authors:** Houda Neani, Hajar Ben Mohimd, Hicham Benyahia, Mohamed Faouzi Azaroual, Fatima Zaoui, Asmae Bahoum, Rajae Elhaddaoui, Loubna Bahije

**Affiliations:** 1 Département d’Orthopédie Dentofaciale, Université Mohammed V, Rabat, Maroc

**Keywords:** Inclinaison de l’incisive, repositionnement incisive, point A, point B, céphalométrie, Incisor inclination, incisor repositioning, point A, point B, cephalometry

## Abstract

**Introduction:**

les points A et B sont des repères squelettiques utilisés dans les études céphalométriques afin d´évaluer les rapports sagittaux entre le maxillaire et la mandibule, respectivement. L´objectif de ce travail est d´évaluer la fiabilité des points A et B comme repères squelettiques en étudiant l´influence du repositionnement incisif sur leur position céphalométrique.

**Méthodes:**

la superposition des tracés céphalométriques de début et de fin de traitement de 30 patients hors croissance, présentant une biproalvéolie, et ayant bénéficié d´un traitement orthodontique avec extraction des quatre premières prémolaires a été réalisée pour évaluer le changement de la position des points A et B. Le seuil de significativité a été fixé à 0,05.

**Résultats:**

dans notre étude, nous avons trouvé que le traitement orthodontique n´influence pas de manière statistiquement significative la position du point A. tandis que le point B est influencé de manière statistiquement significative par la quantité du repositionnement incisif (p= 0,01). En effet pour chaque 1 mm de repositionnement incisif, le point B recule de 0,17mm.

**Conclusion:**

le repositionnement incisif à l´arcade mandibulaire a induit un changement dans la position du point B vers l´arrière.

## Abstract (en)

**Introduction:**

points A and B are bony landmarks used in cephalometric studies to assess sagittal ratio between maxilla and mandible. The purpose of this study is to assess the reliability of points A and B as bony landmarks, by investigating the role of incisor repositioning on their cephalometric position.

**Method:**

superposition of cephalometric tracings at beginning and end of treatment of 30 patients without bone growth disorders, presenting with biproalveolia and having undergone orthodontic treatment with extraction of four first premolars was carried out to estimate changes in points A and B position. The significance threshold was set at 0.05.

**Results:**

our study showed that the influence of orthodontic treatment on point A position was not statistically significant, while its influence on point B position was statistically significant (p= 0,01). Indeed, for every 1mm of incremental repositioning, point B moved back by 0.17mm.

**Conclusion:**

incisor repositioning in the mandibular arch induces a change in the position of point B backwards.

## Introduction

Dans notre pratique quotidienne, les anomalies du sens antéro-postérieur constituent le motif de consultation le plus fréquent du fait de l´importance du déficit qu´elles entrainent. En vue d´obtenir un résultat esthétique et fonctionnel, l´orthodontiste dispose d´un arsenal thérapeutique important allant de l´orthopédie à la chirurgie orthognatique. Le diagnostic positif de ces anomalies du sens sagittal repose d´une part sur les données cliniques, et d´une autre part, sur l´examen radiographique et céphalométrique qui jouent un rôle primordial à la fois diagnostique par la quantification du décalage squelettique et dentoalvéolaire, mais aussi pronostique grâce aux superpositions et les prévisions de croissance qu´ils permettent. Plusieurs mesures sont proposées afin de chiffrer les anomalies du sens sagittal en utilisant les points A et B qui sont des repères squelettiques permettant d´évaluer la relation sagittale entre le maxillaire et la mandibule, à savoir: la projection orthogonale des points A et B sur la ligne SN [1], la projection orthogonale des points A et B sur le plan palatin [2]. La projection orthogonale des points A et B sur le plan occlusal [3]. Les angles SNA et SNB qui sont considérés comme des indicateurs de protrusion des maxillaires [4]. La distance entre la projection orthogonale du Nasion sur le plan Frankfort les points A et B [5].

Toutes ces mesures font appel au point A qui est le point le plus déclive de la concavité du procès alvéolaire maxillaire, et au point B qui est le point le plus déclive de la concavité du procès alvéolaire mandibulaire dans la détermination de la position sagittale des maxillaires. Plusieurs auteurs se sont intéressé à l´investigation de ces deux points et ont mené diverses études dans le but d´évaluer leur stabilité en tant que repères squelettiques n´obéissant qu´aux effets de la croissance cranio-faciale [6]. Ces études reposaient sur différents paramètres d´évaluation du déplacement incisif. En effet, certaines d´entre elles se sont focalisées sur l´inclinaison de l´axe incisif, et d´autres sur le torque incisif, et leurs effets sur la position de ces deux repères. Cette polémique nous a poussé à mener notre propre étude s´intéressant non seulement à l´effet de l´inclinaison de l´axe de l´incisive et le déplacement de son apex, mais également au paramètre de repositionnement incisif, qui a fait l´originalité de notre travail, et son influence sur la position céphalométrique des points A et B.

## Méthodes

**Type et objectif d´étude**: il s´agit d´une étude rétrospective, longitudinale dont l´objectif principal était d´évaluer le changement de la position des points A et B induits purement par les déplacements dentaires (Le point A et B étant les points les plus déclives de la concavité antérieure de l´os alvéolaire maxillaire et mandibulaire respectivement). Ce dernier a été évalué à travers les 3 paramètres suivants: la quantité du repositionnement incisif, Le redressement de l´axe des incisives maxillaire et mandibulaire et Le torque incisif.

**Recrutement des participants à l´étude**: les téléradiographies de profil de début et de fin de traitement orthodontique de 30 patients Marocains ont été sélectionnés à partir des dossiers de patients suivis au service d´orthopédie dento-faciale (ODF) du centre de consultation et de traitements dentaires de rabat CHIS. Nous avons inclus dans notre échantillon uniquement les patients hors croissance avec biproalvéolie ayant bénéficié d´un traitement orthodontique avec extraction des quatre premières prémolaires. Tandis que tous les patients syndromiques, en cours de croissance ou ayant des antécédents de traitement orthodontique ont été exclus.

**Figure 1:**
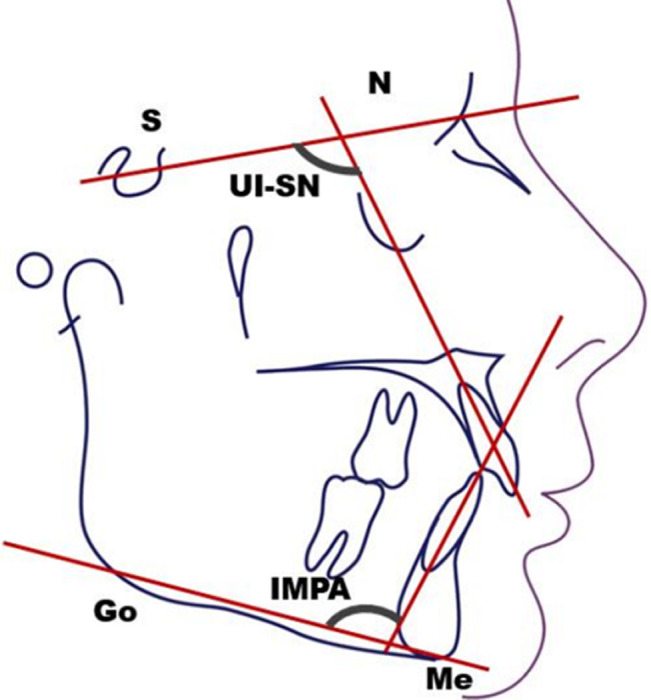
variables angulaires UI-SN et IMPA

**Figure 2:**
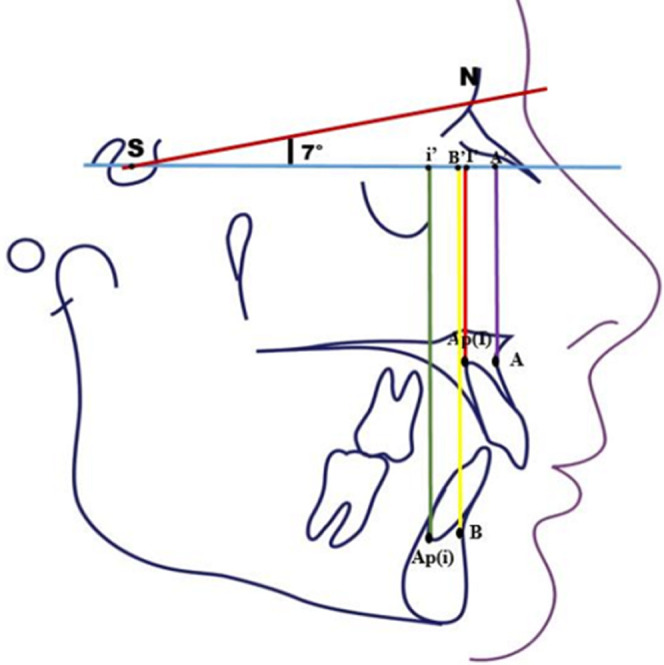
mesures linéaires de la position horizontale du point A, B, Ap(I), Ap(i)

**Figure 3:**
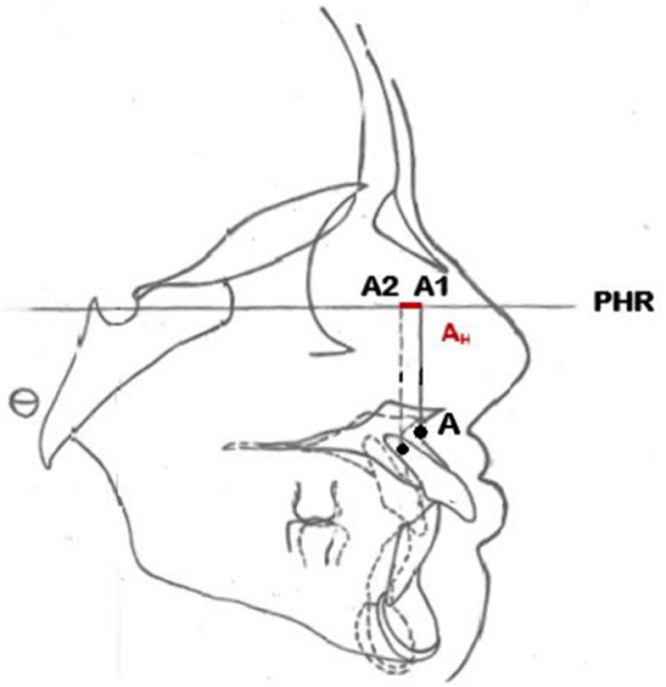
déplacement horizontal total du point A

**Figure 4:**
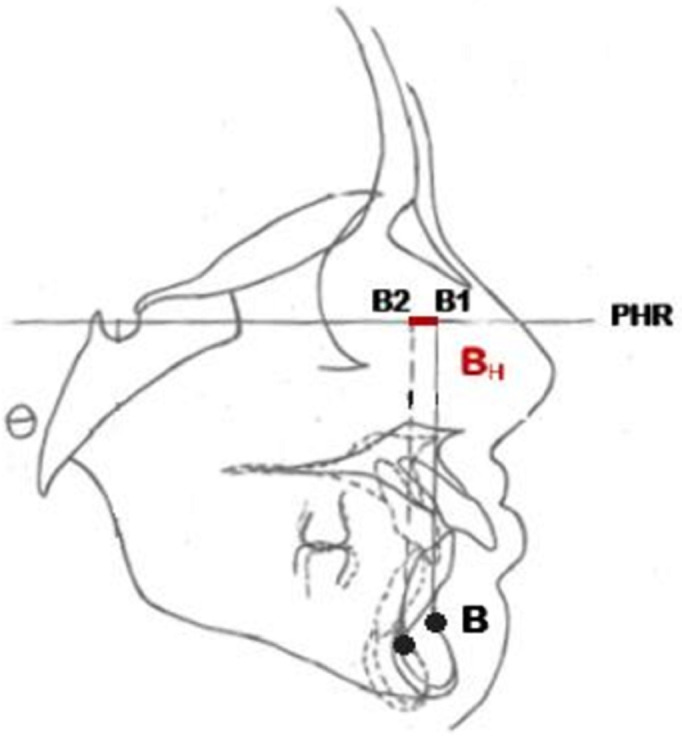
déplacement horizontal total du point B

**Conception de l´étude et mesures utilisées**: après avoir recruté les participants, pour chaque téléradiographie de profil de début et de fin de traitement, nous avons réalisé un tracé céphalométrique et effectué un ensemble de mesures (Tableau 1). Nous avons adopté un plan de référence horizontal passant par le point S et inferieur de 7° de la ligne SN (Figure 1). Pour évaluer l´inclinaison de l´axe de l´incisive maxillaire et mandibulaire nous avons adopté les angles UISN et IMPA respectivement (Figure 2). Pour évaluer la position des points A et B ainsi que celle de l´apex de l´incisive maxillaire et mandibulaire de début et de fin de traitement, nous avons tracé la projection orthogonale de ces points sur le plan de référence adopté (Figure 3, Figure 4). Le déplacement total des points A et B ainsi que celui de l´apex de l´incisive maxillaire et mandibulaire a été évalué à travers la superposition des céphalométries de début et de fin de traitement. La superposition a été réalisé au maxillaire sur la ligne SN avec comme point enregistré le point S, du fait que le point N peut être sujet de certains remodelages relatifs à la croissance nasale. Tandis qu´à la mandibule, nous avons opté pour le contour interne de la symphyse mentonnière vue qu´il s´agit d´une structure stable permettant d´éviter la confusion avec les résultats de la rotation mandibulaire physiologique [7,8].

**Tableau 1:** mesures effectuées sur les tracés céphalométriques

Variables	Signification
UI-SN	Angle formé par l´intersection de l´axe de l´incisive maxillaire et de la ligne Sella-Nasion (SN) et qui matérialise l´inclinaison de l´incisive maxillaire.
IMPA	Angle formé par l´intersection de l´axe longitudinal de l´incisive mandibulaire avec la ligne Gonion-menton et qui matérialise l´inclinaison de l´incisive mandibulaire
Changement UI-SN	Variation de l´angle UISN entre début et fin de traitement
Changement IMPA	Variation de l´angle IMPA entre début et fin de traitement
AH	Déplacement horizontal total du point A.
BH	Déplacement horizontal total du point B.
IH	Déplacement horizontal total de l´apex de l´incisive maxillaire.
IH	Déplacement horizontal total de l´apex de l´incisive mandibulaire.
RI	Quantité du repositionnement incisif

**Figure 5:**
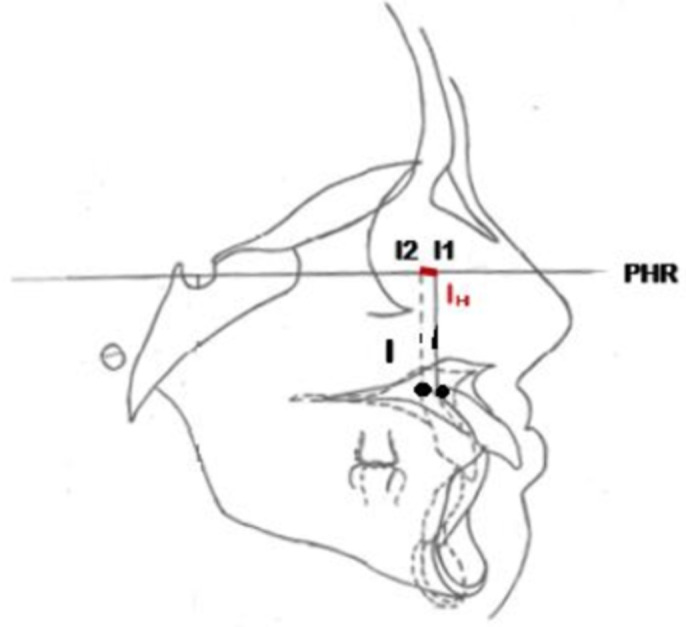
déplacement horizontal total de l'apex de l'incisive maxillaire

**Figure 6:**
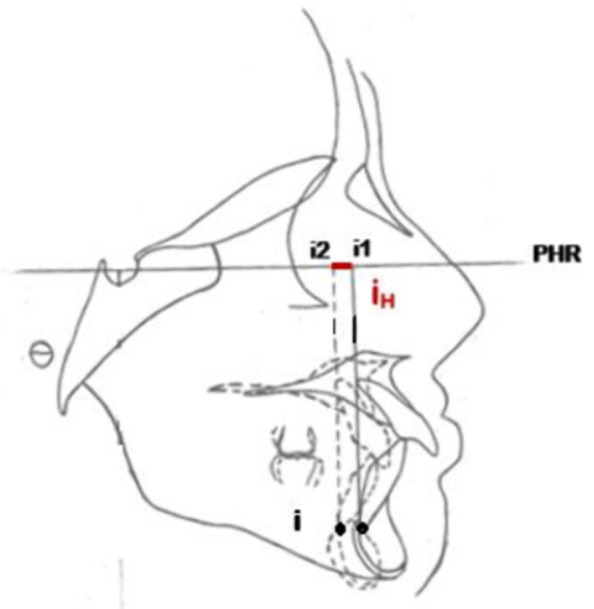
déplacement horizontal total de l'apex de l'incisive mandibulaire

Ainsi, pour évaluer le déplacement horizontal total du point A, nous avons tracé deux lignes verticales A1 et A2, perpendiculaires au plan horizontal de référence et passant par A de début et A de fin de traitement. La distance horizontale AH séparant ces deux lignes verticales matérialise le déplacement horizontal total du point A (Figure 3). De même pour le point B, nous avons tracé deux lignes verticales B1 et B2 perpendiculaires au plan horizontal de référence et passant par B de début et B de fin de traitement. La distance horizontale BH séparant ces deux lignes verticales matérialise le déplacement horizontal total du point B (Figure 4). Avec la même procédure nous avons chiffré le déplacement horizontal total de l´apex de l´incisive maxillaire et mandibulaire (Figure 5, Figure 6). Le signe négatif a été utilisé pour le déplacement vers le haut et vers l'arrière de l´ensemble des points étudiés pendant la collecte des données. En ce qui concerne la quantité de repositionnement incisif, son calcul a été réalisé à partir de la formule de tweed : [FMIA idéale (en fonction du sens vertical)-FMIA (patient)] x 0,8: chez les patients normo (FMIA=68°) et hyperdivergents (FMIA=65°) [IMPA idéale-IMPA (patient)] x 0,8 chez les patients hypodivergents (IMPA=92°) [9].

**Analyse statistique**: l´analyse statistique a été réalisée à travers les logiciels Excel 2007 et SPSS 20.0 Les résultats ont été exprimés en moyenne ± écart-type ou en médiane (Interquartiles) pour les variables quantitatives, et en effectifs et pourcentages pour les variables qualitatives. La comparaison des groupes a été réalisée par le T test pour les groupes appariés et T Student pour les groupes indépendants. Une analyse de régression linéaire multiple a été réalisée pour évaluer la relation de changement de position des points A et B avec les variables restantes, en gardant le changement de position des points A et B comme variables dépendantes et les paramètres restants comme variables indépendantes, alternativement. Le seuil de significativité a été fixé à 0,05. L´analyse de sensibilité demandée a été réalisée par rapport au sexe et par rapport à l´âge, globalement et séparément et nous avons trouvé que notre modèle est robuste, il reste le même et il ne change pas.

## Résultats

L´échantillon que nous avons étudié se composait de 29 sujets (17 femmes: 58,6%; 12 hommes: 41,4%) ayant une moyenne d´âge de 20,90 ± 3,90. 45% des sujets présentaient une classe I squelettique, et 34% une Classe II. La quantité de repositionnement incisif réalisée chez cette population était d´une moyenne de 10,72 ± 5,0. Les tableaux (Tableau 2, Tableau 3, Tableau 4) présentent les valeurs moyennes de chaque variable.

**Tableau 2:** variation thérapeutique des variables angulaires

Variable	Traitement	Caractéristique	P
**SNA**	**DébutFin**	78,76±4,13 79±3,95	0,64
**SNB**	**DébutFin**	74,97±4,13 75,21±3,40	0,66
**ANB**	**Début Fin**	4,21±2,99 3,72±2,17	0,24
**AoBo**	**DébutFin**	2[-2 -3,5]1[-1 -2]	0,78
**FMA**	**DébutFin**	27,31±4,21 26,41±3,76	0,002*
**UISN**	**DébutFin**	106,62±7,26 101,59±7,98	0,002*
**IMPA**	**Début Fin**	95,62±8,56 93,93±6,74	0,19

**Tableau 3:** variation thérapeutique des valeurs linéaires

Variables	Traitement	Caractéristiques	p
Position horizontale du point A	**Début**	40,07 ± 7,39	0,64
	**Fin**	40,38 ±8,04	
Position horizontale de l´apex de l´incisive maxillaire	**Début**	45,72 ± 7,31	0,60
	**Fin**	45,24 ± 8,41	
Position horizontale du point B	**Début**	72 ± 11,92	0,24
	**Fin**	72,55 ± 12,35	
Position horizontale de l´apex de l´incisive mandibulaire	**Début**	78,14 ±10,53	0,78
	**Fin**	76,86 ± 11,92	

**Tableau 4:** description du déplacement total du point A, B et l´apex de l´incisive maxillaire et mandibulaire

Variables	Caractéristiques
**Déplacement horizontal total du point A**	-2[-2–0] *
**Déplacement horozontal total de l’apex de l’incisive maxillaire**	-1[-4-2] *
**Déplacement horizontal total du point B**	-1[-2–0] *
**Deplacement horizontal total de l’apex de l’incisive mandibulaire**	0[-1,5-1] *

La comparaison des variables angulaires entre début et fin de traitement a montré un redressement de l´axe de l´incisive maxillaire (p=0,002) et une réduction du sens vertical statistiquement significatifs (p=0,002) (Tableau 2). La variation des valeurs linéaires intéressant la position des points A, B et l´apex de l´incisive maxillaire et mandibulaire entre le début et la fin du traitement orthodontique étaient statistiquement non significatifs (Tableau 3). En évaluant les facteurs individuellement par analyse uni variée et simultanément par analyse multi variée, nous avons trouvé qu´aucun des paramètres étudiés n´influençaient de manière statistiquement significative la position du point A (Tableau 5). Concernant le point B, en ajustant sur l´axe de l´incisive mandibulaire et la position de son apex, l´analyse multi-variée a révélé qu´il est influencé de manière statistiquement significative par la quantité du repositionnement incisif. En effet, pour chaque augmentation de 1mm de ce dernier, le point B recule de 0,17mm (p=0,01*) (Tableau 6).

**Tableau 5:** facteurs influençant la position horizontale du point A

	Analyse univariée			Analyse multivariée		
	β	IC 95%	P	β	IC 95%	P
**UISN**	-0,05	[-0,14 ; 0,02]	0,17	-0,05	[-0,14 ; 0,03}	0,22
**Déplacement total de l´apex**	0,18	[-0,002 ; 0,40]	0,08	0,18	[-0,04 ; 0,39]	0,10
**RI**	0,002	[-0,14 ; 0,14]	0,57	0,002	[-0,13 ; 0,14]	0,97

**Tableau 6:** facteurs influençant la position horizontale du point B

	Analyse univariée			Analyse multivariée		
	β	IC 95%	p	B	IC 95%	P
**IMPA**	-0,02	[-0,10; 0,06]	0,60	-0,09	[-0,19 ; 0,001]	0,051
**Déplacement total de l´apex**	-0,02	[-0,34 ; 0,29]	0,86	0,06	[-0,23 ; 0,35]	0,67
**RI**	-0,09	[-0,01 ; 0,20]	0,07	-0,17	[-0,04 ; 0,30]	0,01*

## Discussion

L´être humain a toujours été à la recherche de la beauté et s´est intéressé depuis longtemps à l´esthétique faciale qui est influencée de manière importante par la disposition des bases osseuses et des structures sus/sous-jacentes. En effet, un équilibre facial requière nécessairement des rapports harmonieux entre les tissus durs, dentaires et cutanés [10]. En présence d´une proalvéolie, la position antérieure des arcades par rapport à leur base osseuse et au massif facial peut être à l´origine d´un déficit à la fois fonctionnel et esthétique. Le traitement orthodontique aura pour objectif de restaurer l´équilibre facial en agissant sur les structures dentaires. Ce traitement consiste en la réalisation d´un repositionnement incisif le plus souvent après extraction des prémolaires et toujours avec une prise en compte optimale des tissus durs et des tissus mous de la face, du profil, de la dynamique musculaire et de la croissance faciale [11,12]. Toutefois, ces thérapeutiques orthodontiques, induisent non seulement le déplacement des dents en malposition, mais également une réponse dans l´os alvéolaire environnant. Cependant, cette dernière reste difficile à observer [13]. Certains auteurs ont rapporté un changement dans la position des points A et B qui pourrait être induit d´une part, par des modifications d´ordre général résultant des mouvements des maxillaires en rapport avec la croissance antérieure du crâne, et d´autre part par des modifications d´ordre local en rapport avec les remodelages osseux associés à l´inclinaison des incisives lors des traitements orthodontiques [10]. Nous avons donc réalisé une étude dont le but est d´évaluer le changement de la position des points A et B induit purement par le repositionnement incisif. Pour cela, nous avons inclus dans notre échantillon des patients hors croissance, avec biproalvéolie et ayant bénéficié d´un traitement orthodontique avec extraction des quatre premières prémolaires.

Après avoir réalisé les tracés céphalométriques de début et de fin de traitement, et effectué les mesures des paramètres indispensables, nous avons utilisé la technique de superposition pour identifier le changement de la position des points A et B durant le traitement orthodontique. Dans notre étude, nous avons trouvé que le traitement orthodontique n´influence pas de manière statistiquement significative la position du point A. Ces résultats s´accordent avec ceux de Ali Altug *et al*. [14], Al-Nimri *et al*. [15], Erverdi *et al*. [16], et Sohaib *et al*. [6] qui ont rapporté un déplacement horizontal du point A non statistiquement significatif (p>0,05). Cependant, d´autres études ont rapporté un changement statistiquement significatif dans la position du point A en rapport avec les remodelages osseux induits par les thérapeutiques orthodontiques. En effet, Al-Abdwani *et al*. [17] ont trouvé que pour chaque 10° de redressement de l´axe de l´incisive maxillaire, le point A a avancé horizontalement de 0,4mm (p=0,028). Quant à Cangelosi *et al*. [18], ils ont rapporté que pour chaque déplacement en arrière de 3,5mm de l´apex de l´incisive maxillaire, le point A a reculé de 1,7mm, de manière statistiquement significative. Pour ce qui est de la position du point B, notre étude a montré l´absence de déplacement statistiquement significatif par rapport aux paramètres du changement de l´axe et du torque incisif. Résultat qui coïncide avec celui de Sohaib *et al*. [6].

Toutefois, Al-Abdwani *et al*. [17] ont rapporté un changement statistiquement significatif dans la position du point B en rapport avec le redressement de l´axe incisif avec pour chaque redressement de 10° de l´incisive inferieure, le point B se déplace en avant de 0,3mm (p=0,05). En ce qui concerne le paramètre de repositionnement incisif qui a fait l´originalité de notre étude, l´analyse multi-variée a montré un changement statistiquement significatif dans la position du point B. En effet, l´équation de prévision [ Y(dép total du point B)= -2,25- 0,02 (diff IMPA)-0, 02( dep total apex) + 0,17(RI)] a montré qu´en ajustant sur l´axe de l´incisive et la position de l´apex, pour chaque 1mm de repositionnement incisif, le point B recule de 0,17mm. Cette divergence des résultats peut être expliquée par la variabilité des critères d´inclusion et d´exclusion fixés et adoptés dans les différentes études y compris le critère de l´âge, le facteur de croissance, et la nature du traitement orthodontique, avec ou sans extraction. Malgré les résultats de notre étude, cette dernière n´est pas sans limites, à savoir la taille d´échantillon qui reste un peu réduite par rapport à certaines études en raison des critères exigés lors de la sélection des patients pour bien répondre à l´objectif du travail. De plus, nous n´avons pas pu étudier chaque classe squelettique à part pour éviter de restreindre encore plus notre échantillon.

## Conclusion

Malgré les limites de notre étude, on peut conclure que: a) seule la position horizontale du point B a été influencée de manière statistiquement significative par la quantité du repositionnement incisif. Le point A a effectué un déplacement horizontal en arrière entre le début et la fin du traitement. Sauf que ce déplacement est statistiquement non significatif (p>0,05).

### Etat des connaissances sur le sujet

- Un traitement orthodontique induit des modifications au niveau des dents déplacées et au niveau de leurs bases osseuses;
- La croissance cranio-faciale est un autre paramètre qui induit des modifications et des remodelages au niveau du squelette cranio-facial et influence les résultats thérapeutiques lors d´un traitement orthodontique.

### Contribution de notre étude à la connaissance

- Les critères d´inclusion de notre étude incluent uniquement les patients hors croissance afin de limiter les résultats aux déplacements induits purement par les mouvements orthodontique;
- L´analyse du paramètre du repositionnement incisif a montré que la position du point B change de manière statistiquement significative sous l´influence du repositionnement des incisives mandibulaires, contrairement au point A qui ne montre pas de changement statistiquement significatif consécutif au repositionnement des incisives maxillaires.
